# Defining drinking water metal contaminant mixture risk by coupling zebrafish behavioral analysis with citizen science

**DOI:** 10.1038/s41598-021-96244-4

**Published:** 2021-08-27

**Authors:** Remy Babich, Emily Craig, Abigail Muscat, Jane Disney, Anna Farrell, Linda Silka, Nishad Jayasundara

**Affiliations:** 1 Department of Molecular and Biomedical Sciences, University of Maine, Orono, ME 04469 USA; 2 School of Marine Sciences, University of Maine, Orono, ME 04469 USA; 3 MDI Biological Laboratory, Salisbury Cove, ME 04609 USA; 4 Senator George J. Mitchell Center for Sustainability Solutions, University of Maine, Orono, ME 04469 USA; 5 The Nicholas School of the Environment, Duke University, Durham, NC 27708 USA

**Keywords:** Metals, Environmental chemistry

## Abstract

Contaminated drinking water is an important public health consideration in New England where well water is often found to contain arsenic and other metals such as cadmium, lead, and uranium. Chronic or high level exposure to these metals have been associated with multiple acute and chronic diseases, including cancers and impaired neurological development. While individual metal levels are often regulated, adverse health effects of metal mixtures, especially at concentrations considered safe for human consumption remain unclear. Here, we utilized a multivariate analysis that examined behavioral outcomes in the zebrafish model as a function of multiple metal chemical constituents of 92 drinking well water samples, collected in Maine and New Hampshire. To collect these samples, a citizen science approach was used, that engaged local teachers, students, and scientific partners. Our analysis of 4016 metal-mixture combinations shows that changes in zebrafish behavior are highly mixture dependent, and indicate that certain combinations of metals, especially those containing arsenic, cadmium, lead, and uranium, even at levels considered safe in drinking water, are significant drivers of behavioral toxicity. Our data emphasize the need to consider low-level chemical mixture effects and provide a framework for a more in-depth analysis of drinking water samples. We also provide evidence for the efficacy of utilizing citizen science in research, as the broader impact of this work is to empower local communities to advocate for improving their own water quality.

## Introduction

Clean drinking water is at risk due to chemical contamination from both natural and anthropogenic sources. This is a pressing issue in Maine and New Hampshire. Approximately 40% of New Hampshire residents and 40–45% of Maine residents depend on private wells for their drinking water^1–3^. Over one million people across both states are at risk for drinking well water containing more than 10 ppb of Arsenic (As), the maximum contaminant level set for public water systems by the U.S. Environmental Protection Agency (USEPA). Of particular concern, is a high risk of metal contamination in wells in these two states, due to the bedrock geology^3,4^. Neighboring states such as Massachusetts, Vermont, and New Jersey also report high utilization of well water and are at increased risk of chemical contamination^5,6^.

Arsenic contamination of drinking water is an important public health concern in New England as well as around the world. Arsenic is present within bedrock in As-sulfide complexes, and can be easily released into the groundwater supply under alkaline and reducing conditions^7^. Exposure to As has been linked to many diseases including lung, bladder, liver, and skin cancers^8–10^ as well as vascular and neurological disorders^11^. Studies have also linked prenatal As exposure to increased risk of stillbirth^12^ as well as increased As exposure to deficits in intellectual function in adolescents^13^. Considering the adverse outcomes of As exposure, the US EPA reestablished the maximum contaminant level (MCL) of As in drinking water from 50 to 10 ppb in 2001, giving municipalities with public water systems until 2006 to adhere to the new standards.

In Maine and New Hampshire, As remains a critical concern^14^. According to a USGS survey of As in private wells in Maine from 2005 to 2009, the majority of towns contained wells with maximum As concentrations between 10 and 50 μg/L and some exceeding 500 μg/L^15^. A 252 household study in Maine showed that As levels in 1/3 of wells tested exceeded 10 μg/L. Notably, children in households that exceeded 5 μg/L of As were found to have significantly lower IQ scores^16^.

In addition to As, individuals are exposed to other metal contaminants such as lead (Pb), uranium (U), and cadmium (Cd) in the drinking water^17^. Early Pb and Cd exposures have been associated with lower IQs and behavioral disorders in children^18,19^. Gestational exposure to U has shown modifications in locomotor activity and memory in rodent models^20^. The additive or interactive effects of exposure to multiple metal contaminants through the drinking water is beginning to be studied. Importantly, an increasing number of studies show that exposure to chemical mixtures with concentrations below MCLs have biological consequences^21–23^. While integrating mixture impacts in drinking water quality assessment is just beginning to emerge, this is not a consideration at the regulatory level.

Private well water is not mandated by the law to be tested and homeowners are responsible for their own tests. In Maine, many homeowners who have had their wells tested for contaminants in the past have not done so again (even though the recommended testing frequency is 3–5 years) and are often optimistic that their water is clean compared to their neighbors^24^. Even for individuals who are taking action, mitigation methods, such as using a portable water filter or installing a reverse osmosis system, are not always effective, resulting in a false sense of security that can lead to inadvertent metal contaminant exposure^25,26^.

In vivo studies based on model organisms offer a relatively quick and controlled effect-based approach to screening for chemical mixture toxicity of drinking water contaminants, while providing a real-world example for citizen engagement. In particular, the zebrafish (*Danio rerio*), is a prominent high throughput toxicological model^27–29^ with a rapid embryonic development time. Although the zebrafish has been used to assess toxicity of chemicals and chemical mixtures, zebrafish embryos are just beginning to be utilized in deriving biological outcomes from actual drinking water samples^30^.

There are two limitations that are currently impeding the effectiveness of determining drinking water quality. The first that is particularly relevant to Maine and New Hampshire, is a lack of homeowner participation in both water testing and mitigation processes. The second, and a more globally prevalent issue is the lack of information related to metal mixture effects and the difficulty in communicating mixture effects to the broader public.

**Figure 1:**
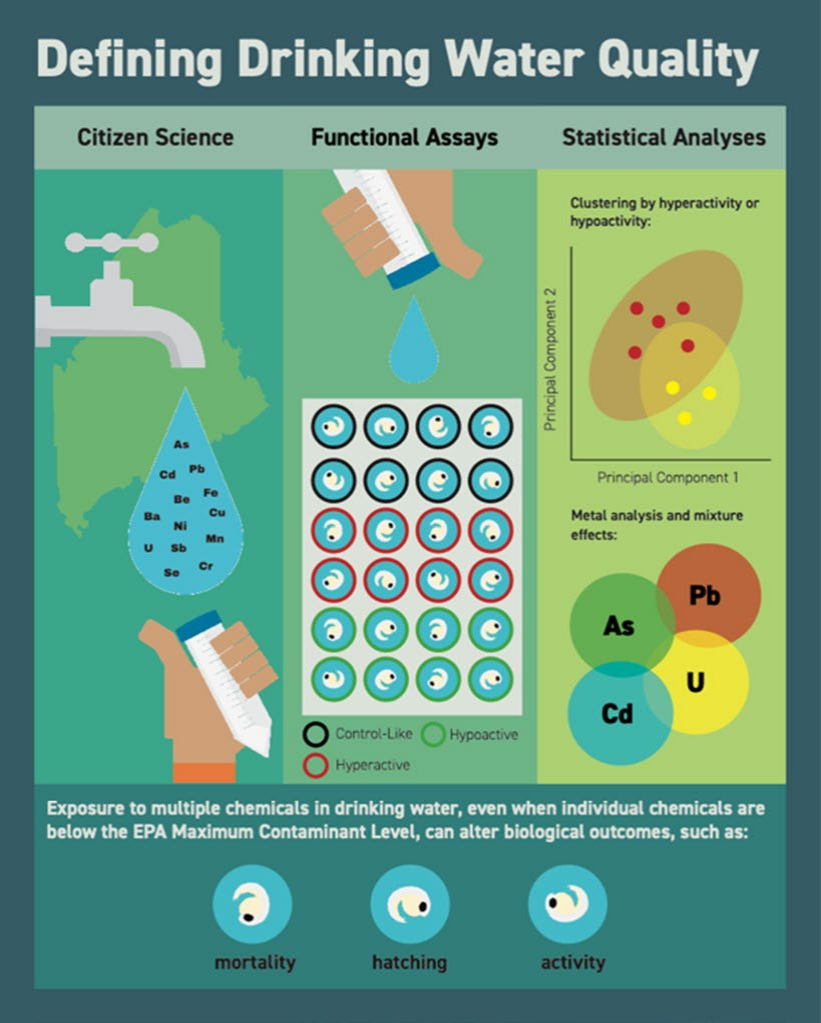
Conceptual map depicting the combination of main methods used in this study including; citizen science, an in vivo functional assay using the zebrafish model, and rigorous statistical cluster analysis. Also highlighted is the main finding, that alterations in biological outcomes (mortality, hatching, and activity) are highly dependent on chemical mixtures even at low concentrations.

Here, given the association of lower-IQ levels in children with arsenic-contaminated drinking water in Maine and other demonstrated effects of arsenic on neurobiology^13,16^, we sought to explore neurobehavioral effects of exposure to drinking water collected from wells in Maine and New Hampshire. To improve homeowner participation in well water testing and increase awareness of physiological effects of metal mixtures, we implemented a citizen science—scientific partner approach, facilitated by teachers in Maine and New Hampshire, who educated and guided students to collect local well water samples for heavy metal analysis. We used these student-collected water samples in blind studies to test behavioral toxicity using the zebrafish model organism to provide a more comprehensive analysis of water quality and potential underlying low-level and mixture effects (Fig. 1).

## Methods

### Outreach and sample collection

Well water samples were collected as part of an NIGMS Science Education Partnership Award (SEPA) project called “Data to Action: A Secondary School-Based Citizen Science Project to Address Arsenic Contamination of Well Water. This collaborative project engages teachers and students from rural schools in Maine and New Hampshire as citizen scientists in collecting well water from their homes for arsenic analysis (Fig. SI). Teachers are recruited through several partnerships and receive training alongside scientist partners.

Well water samples were collected by students from their homes or from neighboring homes or summer camps between spring 2019 and winter 2020. The sampling protocol is as follows: The cold water tap is run for five minutes, after which 50 mL of water is collected in a plastic conical 50 mL tube. The caps to the tubes are wrapped in parafilm in order to prevent leakage when being shipped out for analysis. A second 100 mL water sample is collected from the running tap in a 100 mL sample plastic jar for fish behavior analyses. These samples are frozen for 24 h at the student’s home to kill microorganisms that could set up biogeochemical cycles within the jar and change the speciation of the arsenic^31^. Otherwise, the samples are brought into the classroom and frozen for 24 h by the teacher, who makes note of the time between sampling and freezing. Students collect metadata, often on a paper datasheet, which is later entered into the “All About Arsenic” project on the citizen science data portal Anecdata.org. The metadata include the collector’s name, the student name, the location of the well, the type of well, whether the water was filtered and whether the filter was for the whole home or located at the tap. Information about previous testing is also collected. The name and address of the sampler and exact well location are not available in the publicly available dataset on Anecdata. However, disclosure forms are provided to families so that they can give permission for data to be shared with the Maine Center for Disease Control, New Hampshire Department of Environmental Services, and with scientists conducting research on the well water samples.

### Water analysis

The Trace Element Analysis (TEA) laboratory at Dartmouth College conducts low-level trace metal analysis on student-collected well water samples collected by students using inductively coupled plasma mass spectrometry (ICP-MS) with a triple quadrupole Agilent 8900 (Santa Clara, CA) in helium and oxygen modes. The ICP-MS was calibrated using NIST-traceable standards and calibration was verified using second source standards after the calibration standard and every ten samples. The laboratory control solutions used were NIST 1640a and a USGS proficiency test reference sample. Analytical duplicate and spikes were analyzed at a frequency of one each per 20 samples.

The samples were tested for the following metals: Arsenic (As), Antimony (Sb), Barium (Ba), Beryllium (Be), Cadmium (Cd), Chromium (Cr), Copper (Cu), Iron (Fe), Lead (Pb), Manganese (Mn), Nickel (Ni), Selenium (Se), Thallium (Tl), Uranium (U).

### Toxicity studies

#### Zebrafish exposure

Zebrafish embryos (AB Wildtype) were collected and incubated at 28.5 °C in egg water (60 μg/mL Instant Ocean sea salt in deionized water) at 1 embryo/1 mL until 24 h post fertilization (hpf) and screened for viability. 15 embryos were then moved to treatment solutions containing 7.5 mL egg water and 7.5 mL of a given well water sample (or 15 mL egg water for control exposure) at 28.5 °C until 5 days post fertilization (dpf) in a 30 mL (diameter 6.5 cm, depth 2.5 cm) glass petri dish. 15 embryos were dosed in triplicate, collected from 3 different batches of eggs, per treatment solution. The 50% dilution was used because it was non-lethal but still produced alterations in behavior and allowed the conservation of limited water samples. Of the total 382 samples collected from Maine and New Hampshire, 92 samples were chosen randomly for the behavioral analysis.

#### Behavioral studies

**Figure 2:**
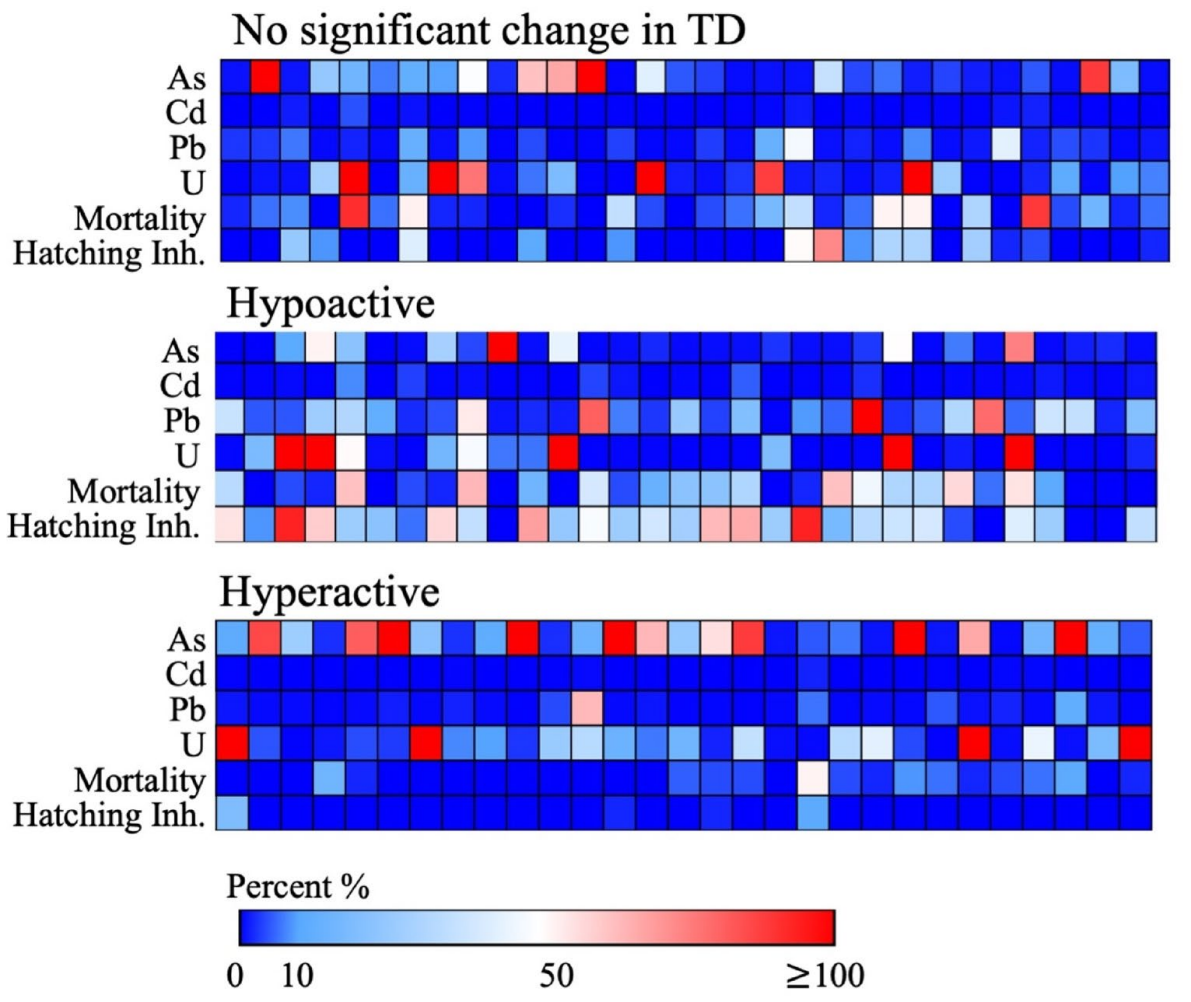
Heat map, representing concentration of a given metal as a percent of its maximum contaminant level (MCL) in a drinking water sample; MCLs for arsenic (As)—10 ppb, cadmium (Cd)—5 ppb, lead (Pb)—15 ppb, and uranium (U)—30 ppb. Percent mortality and hatching inhibition at 5 dpf are also included. Samples are further categorized into exposures that resulted in no significant change in larval total distance (TD) traveled, significant hypoactivity, and significant hyperactivity relative to egg water controls. Blue colors indicate a lower percentage and reds indicate a higher percentage.

Zebrafish larvae were analyzed at 5 dpf for mortality, chorion presence, and any obvious deformities. For a given sample treatment, 8 larvae were randomly selected and placed individually into wells containing 2 mL of egg water each in a 24 well plate. This process was repeated 3 times, yielding behavior data for 24 larvae. There were certain instances where exposure to well water resulted in significant mortality or hatching inhibition. Larvae that remained in their chorion were manually dechorinated and allowed to equilibrate before being plated in 24 well plates, larvae that exhibited minimal movement post-dechorination were not used. In groups with high mortality, extra embryos from the same exposure group were used to obtain a sufficient number of larvae for the behavior analysis. The mortality and hatching data are indicated in Fig. 2 and Supplemental Table SI.

For the behavior analysis, plates containing larvae were kept in a water bath inside Danio Vision (Noldus, Leesburg VA) to maintain temperature at 28.5 °C throughout the run. The Ethovision (Noldus, Leesburg, VA), a software used in tandem with Danio Vision, was used for a light/dark test, which included a 5:00 min dark habituation period, 5:00 min light period, 5:00 min dark period, 5:00 min light period, and 5:00 min dark period (for a total of 25 min). After the run was completed, larvae were removed and euthanized with MS-222. Zebrafish research were carried out in accordance with ARRIVE guidelines and all methods were in compliant with relevant guidelines and regulations and were approved by the University of Maine IACUC committee, proposal number A2017‐05‐04.

### Statistical analysis

The distance traveled by each larvae was extrapolated from Ethovision software at 30 frames/second as mm moved per minute and summed over 25 min to determine the total distance (TD) traveled by a given larvae during the experiment. Averages of TD over 25 min from each larvae were calculated for each treatment group. Prior to statistical analysis, TD data were checked for normal distribution (Shapiro–Wilk), homogeneity of variance (Levenes test), and outliers in SPSS software (IBM, Armonk, NY). To test for statistical significance between TD across sample treatment groups, a one-way analysis of variance (ANOVA) and Tukey post-hoc was conducted using GraphPad Software (Prism, San Diego, CA). This test designated control-like, hyperactive, and hypoactive behavioral groups. Subsequently, ANOVA and Tukey post-hoc was used to test for significance in mortality and chorion presence of samples associated with hypo/hyper/control-like activity relative to egg water controls. Here hypoactive is defined as a sample that results in a significant decrease in TD relative to egg water controls, hyperactive is defined as a sample that results in a significant increase in TD relative to egg water controls, and control-like is defined as a sample that resulted in no significant change in TD relative to egg water controls.

**Figure 3:**
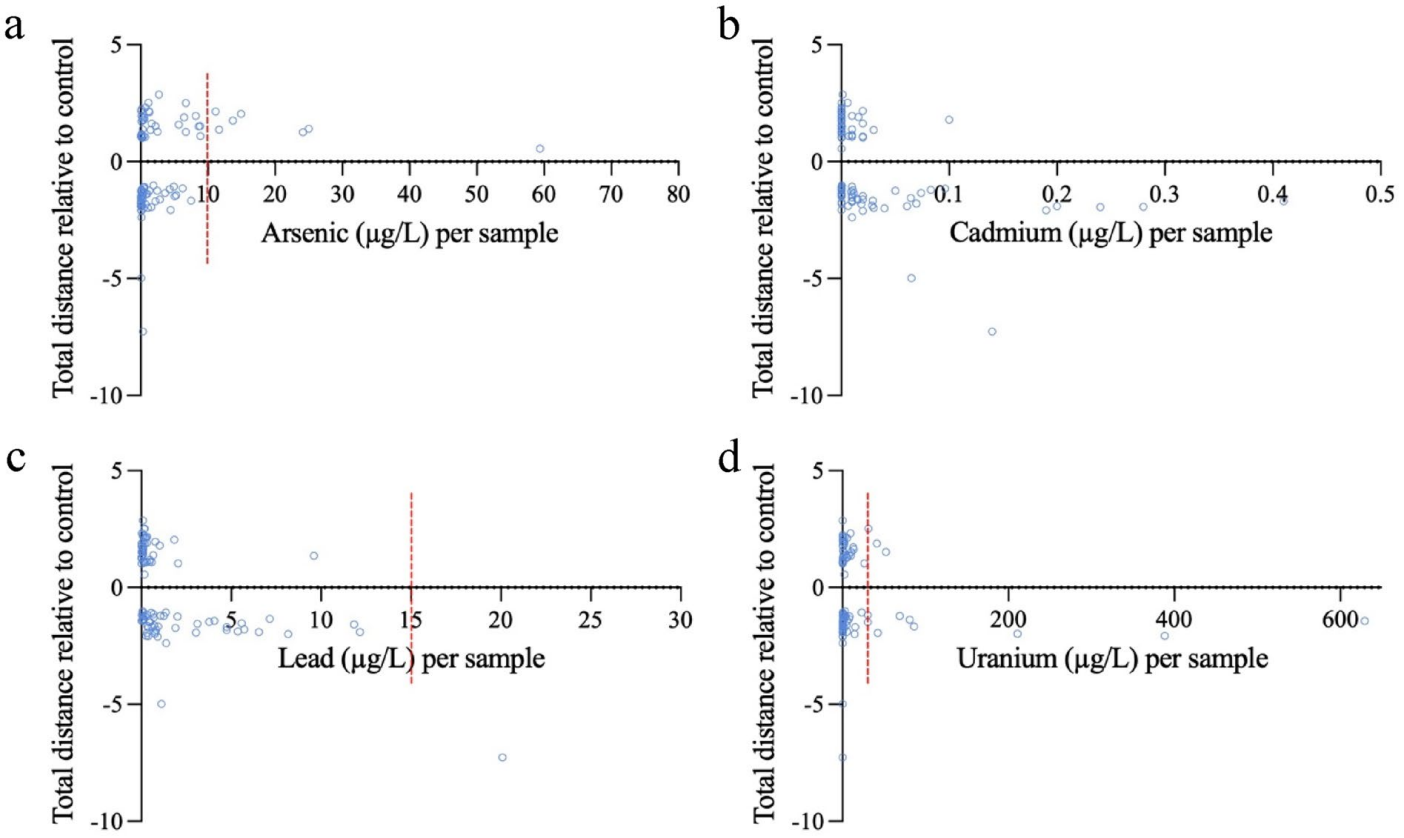
Dot plot representing total distance after a 1:1 sample/egg water exposure. Data is shown in the form of fold change difference between treatment relative to 100% egg water control against the amount of (**a**) arsenic (µg/L), (**b**) cadmium (µg/L), (**c**) lead (µg/L), and (**d**) uranium (µg/L) present in a given sample. Dashed red line represents current EPA maximum contaminant levels in drinking water. Samples that induced significant hyper or hypoactivity can be found in Supplemental Table SI, *p value* < 0.05, ANOVA, n = 24.

To determine the potential for one chemical contaminant to drive the behavioral effects detected following exposure to a given well water sample, a simple correlation analysis was conducted where total distance traveled was plotted against the concentration of a given chemical of interest (Fig. 3, Supplemental Fig. SII). Given previous behavior toxicity studies, we focused on examining a correlation between As, Cd, Pb, and U concentration and larval behavior (Supplemental Fig. SIII).

To evaluate metal mixture effects, we conducted a principal component analysis (PCA) using SPSS software (see supplemental methods for syntax, IBM, Armonk, NY). The PCA generates 2 principal components (PC1, PC2) for each of the 92 well water samples based on their chemical composition, which was inserted into the PCA as raw concentration values in μg/L. The PCA can be performed based on all 12 chemical composition data or user picked combination of chemicals (e.g., PCA can be conducted based only on As, Cd, and Pb concentration data to determine principal components for all 92 wells). Given there are 12 metals, numerous combinations of metal concentration data, can be integrated into the PCA. To determine the number of combinations of metals (i.e. different permutations of As, Sb, Ba, Cd, Cr, Cu, Fe, Pb, Mn, Ni, Se, and U combinations, from a mixture containing all 12 down to that of three metals remaining in each combination)

1 $$\Sigma \;n!/r!\left( {n - r} \right)!$$

here n = 12 and r is all integers between and including 11 and 3, resulting in a total of 4016 combinations without repetition. (Although Be and Tl levels were determined in the metal analysis, they were excluded in subsequent mixture analyses, since they were undetected in the majority of well samples tested). PC1 and PC2 for all possible 4016 combinations were generated.

PC1 and PC2 for each of the 92 samples were then plotted for each of the 4016 combinations. Each of the 92 samples within a PCA plot was then overlayed with behavioral data (i.e. designated as hyperactive, hypoactive, or control-like behavior). This allowed for visualizing clusters of behavior among the 92 sample treatments, based on the metals and their corresponding concentrations for a given metal input combination. Subsequently, we focused on metal combinations that resulted in distinct clustering between groups of control-like and hypoactive associated samples or control-like and hyperactive associated samples.

To reduce ambiguity in determining different clusters, we utilized an approach as demonstrated by Goodpaster and Kennedy^32^ to identify statistically significant clusters between different behavior response groups for a given metal combination and repeated it for all 4016 possible metal combinations. Briefly, PC1 and PC2 were used to generate the Mahalanobis distance (MAH) between groups based upon activity level, e.g., MAH between control-like cluster and hypoactive cluster. Using Excel (Microsoft, Redmond, WA) the MAH was derived from $\surd$d′C_w_^−1^d, where d_average_′ = PC1_(control-like)_ − PC1_(hypoactive)_ and d_average_ = PC2_(control-like)_ − PC2_(hypoactive)_. C_w_^−1^ is the inverse of the pooled variance and covariance matrices between control-like and hypoactive groups. The MAH was then used to determine the Hotteling’s two-sample T^2^ statistic using T^2^ = (n_1_n_2_)/(n_1_ + n_2_)(MAH). Upon calculation of the T^2^ statistic, an F-value = (n_1_ + n_2_ − p − 1)/p(n_1_ + n_2_ − 2)T^2^, could be calculated and used in the F-test. Here, n_1_ = # of well samples associated with control-like activity, and n_2_ = # of well samples associated with hypoactivity, and p = 2 (comparison between 2 clusters). The F-test was then used to determine significance between 2 activity clusters generated via PCA derived principal components. The clusters were considered significantly different if the calculated F-value was greater than the critical F-value as determined by a web-based software (https://www.danielsoper.com/statcalc/calculator.aspx?id=4). The F-critical value for control vs hypo and hyperactive clusters was calculated to be 5.81 and 5.79 respectively, with an alpha level of 0.005.

Statistically significant clusters of control-like and hypoactive or control-like and hyperactive groups were determined for all 4016 metal combinations. From this, we generated two subsets of combinations, those combinations that resulted in significantly different clusters between control-like and hypoactive behavior and control-like and hyperactive behavior. For each subset, the number of times an individual metal appeared within a combination was calculated. This was conducted to determine the prevalence of metals that resulted in distinct hypoactive and hyperactive clusters compared to control-like clusters.

Further, to identify if 2 or 3 metals consistently appeared together within metal combinations of the 2 subsets, similar to earlier, the number of permutations to be tested were first calculated using $\Sigma$ (*n*!/*r*! (*n* − *r*)!). Here, n = 12 and r is 2 or 3. This resulted in 286 tri and bipartite combinations, which were subsequently identified within combinations comprising each subset.

## Results

### Metal analysis

Metal analysis revealed a heterogenous metal composition across the well samples based on the 14 chemical panel that was tested. Corresponding concentrations per each metal from each sample can be found in the supplemental material (Tables SI, SII).

Given previous data on zebrafish behavioral effects of exposure to As, Pb, Cd, and U (Fig. SIII), first, we focused on levels of these metals in our well water samples (Fig. 2). The minimum and maximum As concentrations found among the 92 samples were 0 and 717.9 μg/L respectively with a median of 0.52 μg/L. Eight samples (487, 490, 501, 514, 515, 895, 950, 1238) exceeded the MCL of 10 μg/L (Table SI). The maximum level of Cd was 0.41 μg/L from sample 508, with a minimum of 0 and median of 0.007 μg/L. The maximum level of Pb was 20.1 μg/L from sample 907, and was the only sample to exceed the lead MCL of 15 μg/L. The minimum concentration was 0.01 μg/L and the median was 0.36 μg/L (Table SI). Uranium concentrations ranged from 0 to 3274.37 μg/L with a median of 1.0 μg/L. Twelve of the 92 samples exceeded the MCL for uranium of 30 μg/L (Table SI), while 21 out of the 92 samples contained either As, Pb, or U exceeding the MCLs. The remaining 77% of samples contained levels of As, U, Pb, and Cd considered safe in drinking water (Fig. 2).

### Behavioral toxicity: correlations with individual chemical concentrations

Zebrafish exposed to a 1:1 well water sample to egg water solution resulted in altered TD traveled over a 25 min light dark test period in 60 of the 92 wells tested. Light dark tests have been used extensively in toxicity studies to estimate behavioral effects of contaminant exposure^33–35^. 31 samples induced a significant decrease in activity and 29 induced a significant increase in activity relative to TD traveled of embryos reared in egg water. 32 of the 92 samples showed no significant change in TD compared to egg water controls (Table SI).

To examine potential correlations between known behavior toxicants, individual chemical concentrations of As, Pb, Cd, and U were plotted against TD as a percent of egg water controls (Fig. 3). Eight out of the 92 samples tested contained As above the MCL of 10 μg/L, five of which (11.2, 11.7, 13.7, 14.9 and 25.0 μg/L,) were associated with samples that induced hyperactivity (Fig. 3a). Sample 187, with the highest concentration of As, at 717.9 μg/L did not significantly change TD traveled compared to controls. Additionally, from 10 of the samples containing arsenic levels between 5 -10 μg/L, four were associated with hyperactivity. Low-levels of As in the remaining samples associated with hyperactivity ranged from 0.05 to 2.7 μg/L, the highest concentration of As in all 33 samples associated with hypoactivity was 59.4 μg/L. All other samples associated with hypoactivity contained a range of As from 0 to 7.4 μg/L (Fig. 3a, Table SI).

Cadmium was low among all samples regardless of the associated activity level. The maximum concentration of Cd detected was 0.41 μg/L and was associated with a sample that produced hypoactivity. Well water samples with Cd ranging from 0.14 to 0.28 μg/L, aside from one sample that produced control-like behavior at 0.24 μg/L, were associated with hypoactivity, suggesting a potential connection between Cd exposure and decreased behavior (Fig. 3b).

One well sample contained Pb over the MCL (20.1 μg/L). Including this sample, a total of ten samples contained Pb over 5 μg/L, and seven were associated with hypoactivity (Fig. 3C). The remaining 24 samples associated with hypoactivity had a range of 0.03–4.75 μg/L Pb. The largest concentration of Pb found in those samples producing hyperactivity was 9.6 μg/L with a range of 0.01–1.8 μg/L for the remaining (Table SI).

Presence of U in the drinking water was also associated with behavioral effects (Fig. 3d). Thirteen (30.42–3274.4 μg/L) of the samples exceeded the MCL for U of 30 μg/L, and five (30.42–629.4 μg/L) were associated with hypoactivity in zebrafish. The highest concentration of U was found in a sample associated with hyperactivity at 3274.4 μg/L along with 4 other samples containing concentrations over the MCL (31.1, 41.5, 52.5, and 3274.3 μg/L). Control-like behavior also contained 4 samples exceeding the MCL (30.4, 42.6, 69.1, and 81.0 μg/L). 16 samples contained U over 5 μg/L, these included 4 producing hypoactivity (5.6–15.4 μg/L), 7 producing hyperactivity (5.5–13.6 μg/L), and 5 producing control-like behavior (5.6–26.3 μg/L). The remaining concentrations among all 3 activity levels collectively ranged from 0.01 to 4.9 μg/L (Table SI).

Overall, individual As, Pb, Cd, and U metal levels did not explain behavioral effects detected following exposure to a given well water sample, suggesting a potential role for interactive effects of multiple metals.

### Behavioral toxicity: correlations accounting for metal mixture effects

**Figure 4:**
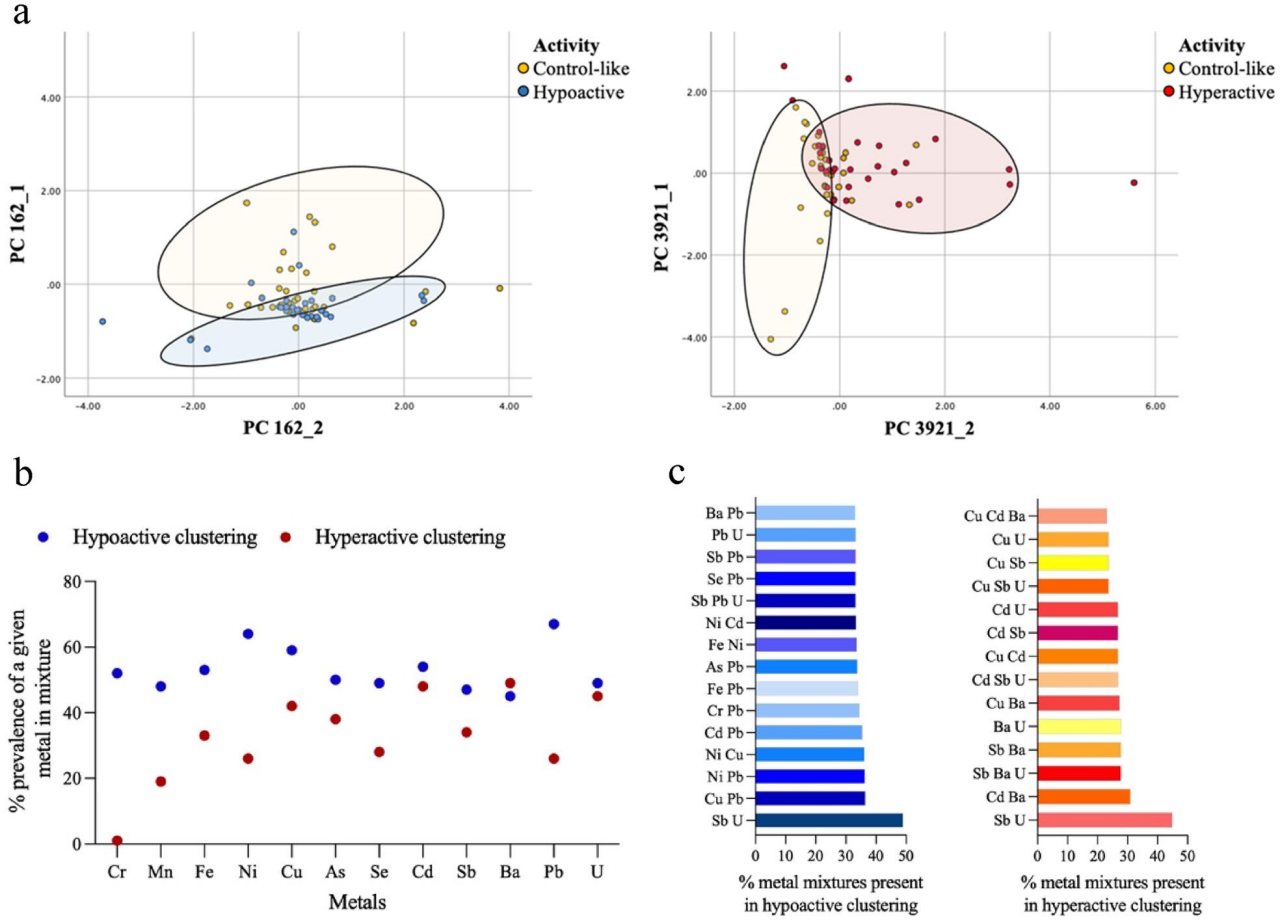
(**a**) Examples of principal component analysis (PCA) derived scatter plots that show significant clustering between control-like and hypoactive and control-like and hyperactive behaviors from metal combination inputs of Cr, Mn, Fe, Se, Cd, Sb, Ba, Pb, & U (combination 162) and Fe, Ni, Cu, Se, Pb, & U (combination 3921) respectively. (**b**) Dot plot representing the percent prevalence of a given metal in mixtures that resulted in significant clustering between control-like and hyperactive (red) as well as control-like and hypoactive (blue) behavior. (**c**) Bar graphs representing the top 15 combinations of two or three metals within mixture subsets that resulted in significant clustering between control-like and hypoactive and control-like and hyperactive behavior. Clusters were created via PCA and an F-statistic was calculated to determine significance, *p* value < 0.005.

Principal component analysis was used to extrapolate a total of 3,982 principal component pairs derived from 4016 chemical combinations as inputs (the statistical software was unable to calculate PC pairs from 34 combinations). Behavioral activity was overlayed on top of principal components to determine if activity level clustered based upon what chemicals and their corresponding concentrations were present in the water samples. Examples of PCA derived scatter plots from a combination of Cr, Mn, Fe, Se, Cd, Sb, Ba, Pb, & U and Fe, Ni, Cu, Se, Pb, & U, that resulted in significant clustering between control-like and hypoactive and control-like and hyperactive behavior respectively, are provided in Fig. 4a. A subset of 2,777, out of the 3,982 tested, metal mixtures resulted in distinct clusters of samples that produced control-like behavior and hypoactivity. Metals that appeared the most within the 2,777 combinations included Pb (67%), Ni (64%), Cu (59%), and Cd (54%) (Fig. 4b). A second subset of 193 metal combinations, resulted in distinct clusters of samples that produced control-like behavior and hyperactivity. Ba, Cd, U, and Cu were present in 49%, 48%, 45%, and 42% of the 193 combinations, respectively (Fig. 4b).

Bipartite and tripartite combinations, containing either 2 or 3 metals, (286 total combinations) were also checked for frequency among the subsets of metal combinations that resulted in significant clustering between control-like samples and those that produced hypo or hyperactivity. The top bipartite combination for both activity trends was Sb and U which was present in 49% of hypoactive and 45% for hyperactive associated subsets (Fig. 4c). Six of the top 15 bipartite and tripartite combinations associated with hyperactivity contained Cd, and 10 of the 15 associated with hypoactivity contained Pb (Fig. 4c).

**Figure 5:**
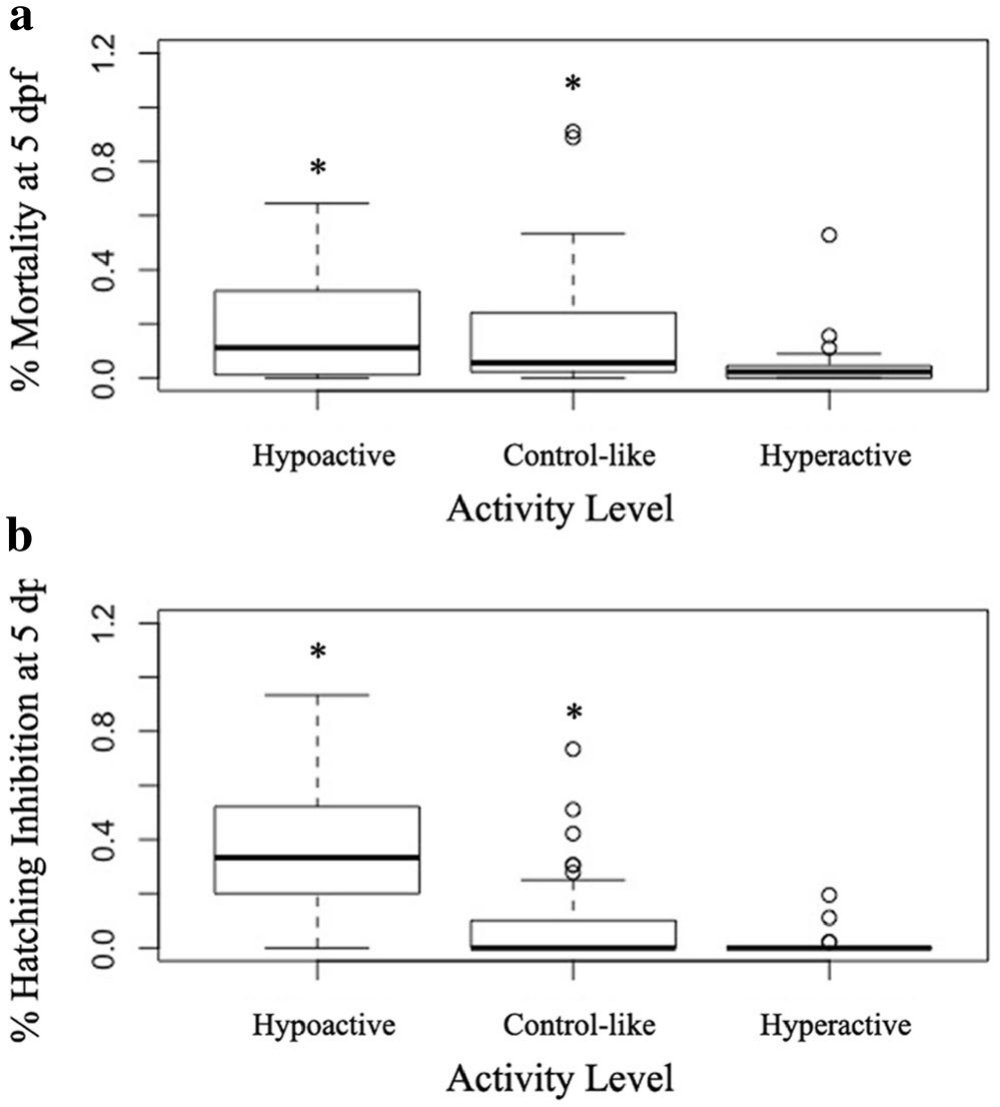
Box plot representing (**a**) % mortality and (**b**) % hatching inhibition at 5dpf after a 1:1 sample/egg water exposure relative to 100% egg water control. Data is plotted based upon samples that significantly induced hypoactivity, hyperactivity, or no significance from egg water controls. Additional information regarding specific samples that induced significant hyper or hypoactivity can be found in Table S1, *p value* < 0.05, ANOVA, n = 24.

Samples associated with hypoactivity and control-like behaviors had a significant increase in % mortality (20%, 17%) and hatching inhibition (35%, 10%) at 5 dpf relative to egg water controls (1.7%, 0%, Fig. 5a,b). There was no significant difference in mortality and hatching inhibition in those samples associated with hyperactivity (4.6%, 1.2%) compared to egg water controls (Figs. 2, 5a,b).

## Discussion

In this study, we present a zebrafish-based toxicity analysis in determining behavioral effects of exposure to metal mixtures in the drinking water. The study was significantly enhanced by utilizing a citizen science approach that enabled collection of a large set of well water samples from families in Maine and New Hampshire. Teachers and students involved in this study gained knowledge in the following areas: health risks due to metal exposure, methods for sampling well water, submitting metadata associated with the well water sample, analyzing the data using a data literacy software called Tuva, and public outreach about the well water results. In this way, we are sharing knowledge and preparing future citizens so that they may generate community awareness around water quality and demand better water quality monitoring standards.

This approach increased our likelihood of obtaining an increased number of well water samples across Maine and New Hampshire, while engaging local teachers and students to discuss the importance of clean drinking water. With our set of 92 samples, we show that although samples may be considered safe with most chemicals at concentrations below the MCLs, there are still potential adverse behavioral impacts. These results were shared with teachers in an annual training workshop. We developed an analogy-based video on PCA analysis for teachers and engaged them in an interactive discussion of our research results.

The results of our study demonstrate that adverse biological outcomes of environmental exposures, such as the behavioral effects detected in the current study, can result from exposure to multiple chemicals, even when individual chemicals are at low to moderate levels. Additionally, a subset of samples do not have an impact on behavior, as seen in our control-like group, which may be due to opposing modes of action of metal contaminants and further highlights the role of mixture and concentration specific effects^36^. Our results are consistent with other emerging studies, including in zebrafish, which show chemical mixture effects on development as well as behavior^37,38^.

Our cluster analysis supports the hypothesis that a single chemical is not driving the changes in zebrafish activity levels, but rather a result of a mixture effect (Fig. 4). By utilizing PCA we demonstrate that distinct clusters of samples that are associated with hypo or hyperactivity still occur, regardless if the metals within the mixtures exceed the MCL. Interestingly, Cd is in the top four metals that are present within both hypo and hyperactive clusters when compared to control-like behavior. In these instances, Cd concentrations remain well below the MCL of 5 ppb (Fig. 4b). Cd has been shown to be toxic when in mixture; for example, in *C. elegans* toxicity increased when exposed to Cd and Cu in tandem^39^. Although concentrations of Cd are lower than the MCL in our well water samples, Cd is in 7/15 of the top bipartite and tripartite combinations present in the subset of metal combinations associated with distinct hyperactive clustering, one of which is Cd and Cu (Fig. 4c).

Along with Cd, Pb was found to be present in 67% of the subset of mixtures that resulted in significantly different clusters between hypoactive and control-like behavior (Fig. 4b). Lead was also present in 10/15 most prevalent bipartite and tripartite combinations associated with distinct hypoactive clustering (Fig. 4c). However, both epidemiological and rodent studies suggest that Pb is more likely to cause hyperactivity at levels of 14.3 μg/L in cord blood and 50 mg/L in drinking water^40,41^. Interestingly, a study subjecting maternal zebrafish to 20 μg/L Pb found that larval offspring in the next generation also showed hyperactivity relative to controls. However, when the maternal generation was exposed to a mixture of 20 μg/L Pb and 5 μg/L crude oil, TD traveled by larval offspring significantly decreased^42^, suggesting that transgenerational behavioral effects of Pb are mixture specific. The concentrations examined in these studies are higher than what was detected in Maine and New Hampshire well water, suggesting that possible interactions with other metals and potential chemicals in the water sample can alter zebrafish behavior.

Uranium was also present in the top four metals that appear in 45% of the subset of mixtures that resulted in significantly different clusters between hyperactive samples verses control-like (Fig. 4b). Uranium, in combination with antimony, is the most prevalent bipartite combination among both hyperactive and hypoactive associated mixture subsets. It is also present in 7/15 top bipartite and tripartite combinations present in the subset of mixtures responsible for hyperactive clustering (Fig. 4c). Specifically, in the zebrafish model, U at concentrations of 20 and 250 μg/L has been found to significantly delay hatching as well as decrease larval body length compared to controls^43^. In addition, U exposure at 100 μg/L has been linked to muscle tissue disruption in adult zebrafish in the form of degenerated myofibrils and abnormal mitochondrial localization^44^. A 10 day U exposure at 250 μg/L has also been shown to damage adult male zebrafish sensory organs, specifically the lateral line and olfactory systems^45^. Although more studies are needed on the impacts of U on development along with behavioral consequences, these data from previous studies may explain the changes in activity associated with U seen in this study.

Arsenic, which is a relevant metal of concern in New England, did not show any obvious trends regarding concentration or presence in samples associated with hyperactivity, hypoactivity, or control-like behavior (Fig. 3a). However, when determining prevalence in mixtures accounting for distinct clusters, As was present in 50% and 38% of mixtures associated with hypo and hyperactivity respectively. It was also higher in prevalence over 5 (Mn, Se, Sb, Ba, U) and 7 (Mn, Fe, Ni, Se, Sb, Pb) other metals in the panel for hypo and hyperactivity associated mixture subsets. Arsenic that was present in mixtures was found at low levels, with the majority of concentrations below the MCL of 10 ppb. These data, in combination with previous epidemiological and model organism studies, emphasizes the need to address the potential toxicity of low levels of As, both individually and in mixture, especially in the contexts of drinking water. For example, it was found that women who have had trouble conceiving in the past, that were exposed to As at 1 μg/L in their drinking water had a significant decrease in likelihoods for pregnancy^46^. Another study showed that a median of 1.2 μg/L As in maternal drinking water correlated with decreased birth weight in smaller infants^47^. Further, it was found that zebrafish exposed to environmentally relevant levels of As (50 and 500 ppb) caused a more severe change in activity when in a zinc-deficient mixture compared to those supplemented with zinc, suggesting that As mediated toxicity is highly dependent upon the presence or absence of other chemicals^21^. Given that we show As is an important factor in mixture toxicity, even at levels ranging from 0.03 to 5.1 μg/L, low levels of As, between 1 and 5 μg/L, should be considered when determining water quality standards.

It should also be observed that the prevalence of some metals, in the subsets of mixtures resulting in distinct hypo or hyperactive clusters, change dramatically based upon activity level. For example, Cr is seen in 52% of mixtures associated with hypoactivity and 1% of mixtures associated with hyperactivity. Pb and Ni are also found in many of the mixtures associated with hypoactivity (67% and 64%) compared to hyperactivity (26% and 26%). This supports the notion that biological endpoints are highly sensitive to metal mixtures and their corresponding concentrations and that the mechanism of action can potentially change given what is present in a mixture.

Further, there is a clear link between activity, mortality, and hatching inhibition at 5 dpf. Mortality, as well as hatching inhibition, after exposure to samples that induced hypoactivity significantly increased compared to egg water controls and those samples that resulted in hyperactivity (Fig. 5). This phenotypic response is indicative that other developmental consequences may be occurring in response to mixture exposure besides neurobehavioral effects. Additionally, changes in phenotype may be contributing to changes seen in behavior^48^. Broadly, our findings indicate a necessity to consider potential developmental impacts of all metal contaminants in drinking water supplies, even at levels considered safe for human consumption.

The zebrafish has been used to test toxicity of many environmental contaminants, such as As and U, during early development^49–51^. It has also been a model, alongside rodent species, for evaluating neurotoxic effects and later in life consequences of heavy metals^52,53^. While zebrafish are established as a relevant toxicological model ^49–52^, certain parameters such as exposure time and route of exposure during embryogenesis are variable compared to human fetal exposure^54^ and should be taken into consideration when drawing conclusions. Nonetheless, we believe data collected using the zebrafish model is highly informative and research combining water chemistry and epidemiological studies followed with multivariate analyses, such as described here, can provide key insights into adverse health outcomes of chemical mixture exposure.

Through complementing water analyses with behavioral outcomes we have shown here that chemicals such as As, Cd, Pb, and U, even at low-levels, should be taken into special consideration when evaluating results from regular water testing. More importantly, we emphasize that other metals that are not typically associated with adverse effects (e.g., Ni and Sb) should be considered in risk assessment when in mixture with chemicals such as Pb and U. Determining safe levels of these chemicals as well as potential mixture effects warrant further study and should be examined when implementing federal regulations. Our study has merged citizen science and toxicology research lending itself to the development of improved testing and assessment methodologies, and to better inform households in Maine and New Hampshire, as well as policy makers, of their local quality of water. Through our synergistic efforts with local schools, we are able to inform teachers and students about new ways to assess water quality and generate engaging and in-depth discussion on chemical mixture effects through fish behavior visualizations following well water exposure.

## Supplementary Information

Supplementary Figures.

Supplementary Table S1.

Supplementary Table S2.

Supplementary Information 1.

## Data Availability

The water chemistry datasets generated during and/or analyzed during the current study are available in the Supplemental Material Tables S1 and S2. Additional behavioral data generated during and/or analyzed during the current study are available from the corresponding author on reasonable request.
