# Supplementary material for: Defining drinking water metal contaminant mixture risk by coupling zebrafish behavioral analysis with citizen science
Source: Sci Rep. 2021 Aug 27;11:17303. doi: 10.1038/s41598-021-96244-4 (PMC8397788; doi:10.1038/s41598-021-96244-4)
Supplement: Supplementary file 1 — Supplementary Figures. [file 41598_2021_96244_MOESM1_ESM.docx]

**Defining Drinking Water Metal Contaminant Mixture Risk by Coupling Zebrafish Behavioral Analysis with Citizen Science**

*Remy Babich^1^, Emily Craig^2^, Abigail Muscat^2^, Jane Disney^3^, Anna Farrell^3^, Linda Silka^4^, Nishad Jayasundara^5^

**Affiliations:**

^1^Department of Molecular and Biomedical Sciences, University of Maine, Orono, ME

04469 US.

^2^School of Marine Sciences, University of Maine, Orono, ME 04469 US.

^3^MDI Biological Laboratory, Salisbury Cove, ME 04609 US.

^4^Senior Fellow, Senator George J. Mitchell Center for Sustainability Solutions,

University of Maine, Orono, ME 04469 US.

^5^The Nicholas School of the Environment, Duke University, Durham, NC 27708 US.

**Figure SI**

*
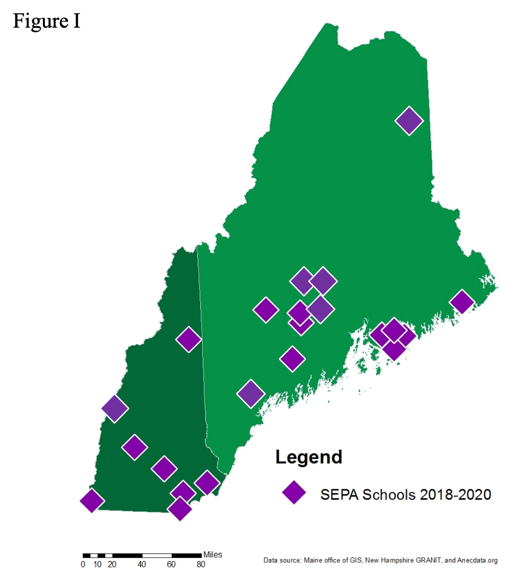
*

Figure SI: Map of Maine and New Hampshire depicting schools (purple diamonds) involved in the SEPA program. Five schools from Maine and 3 schools from New Hampshire contributed drinking water samples used in this study.

**Figure SII**

**
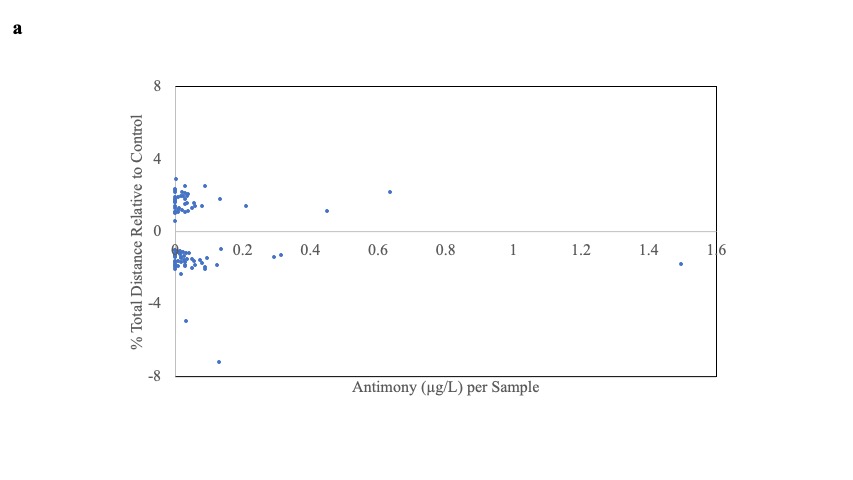

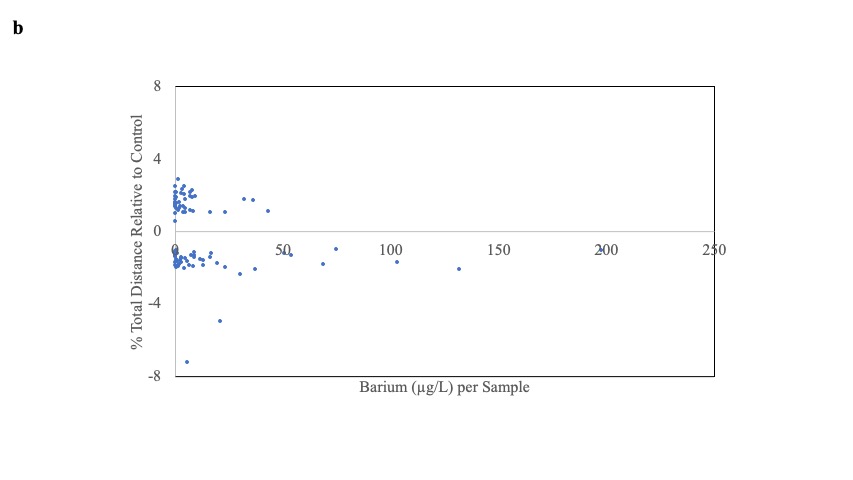

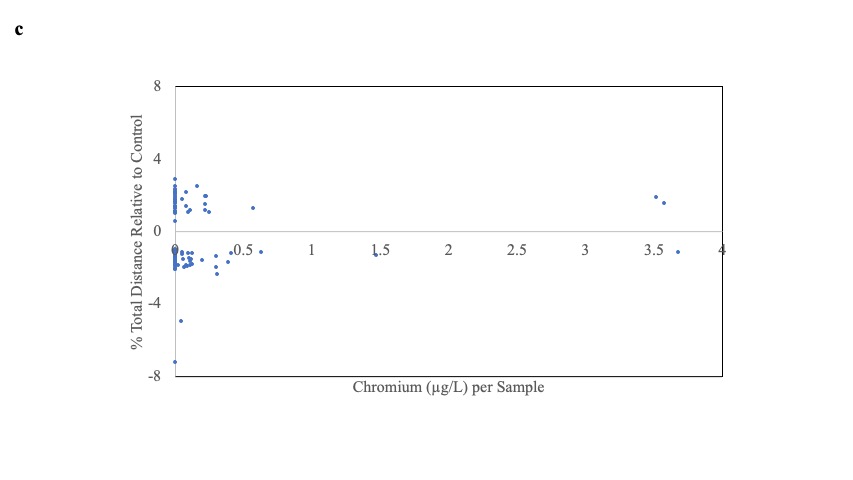

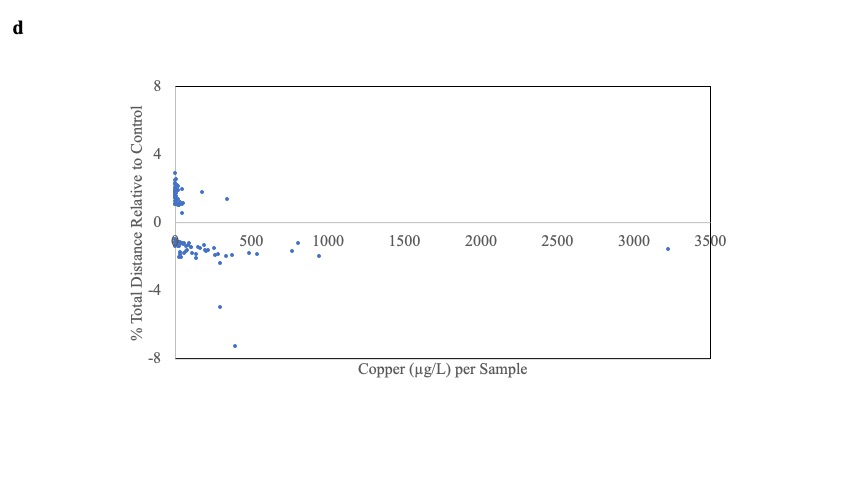

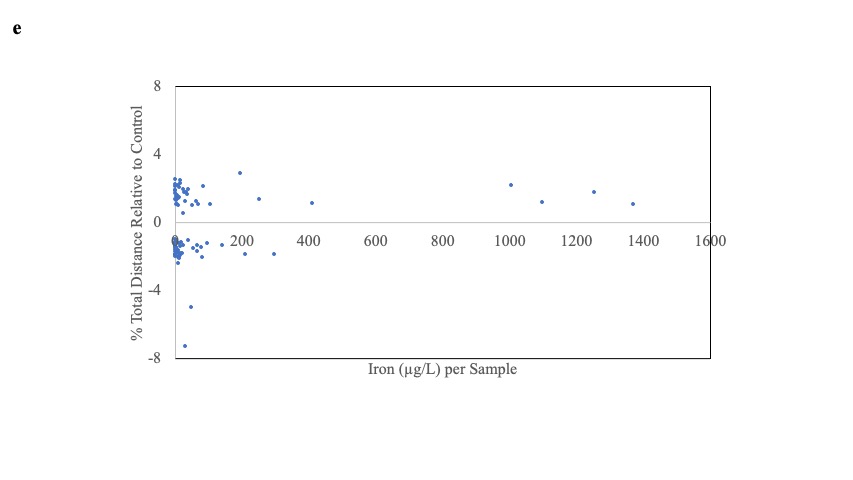

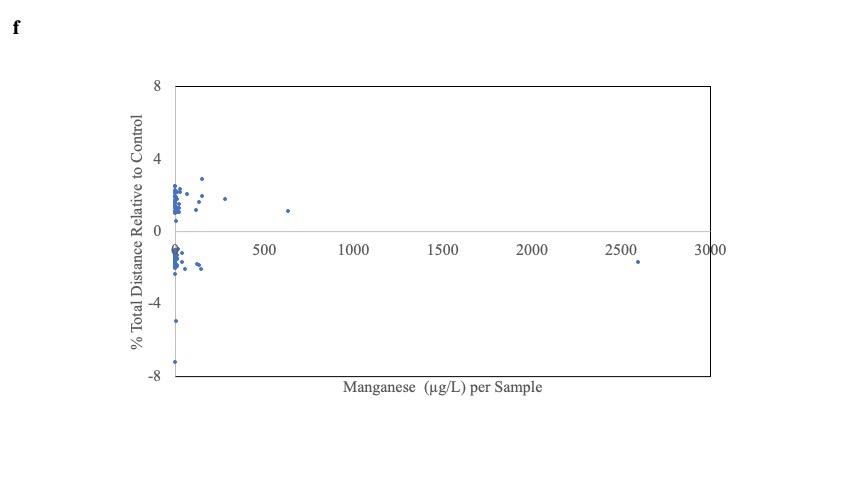
**

**
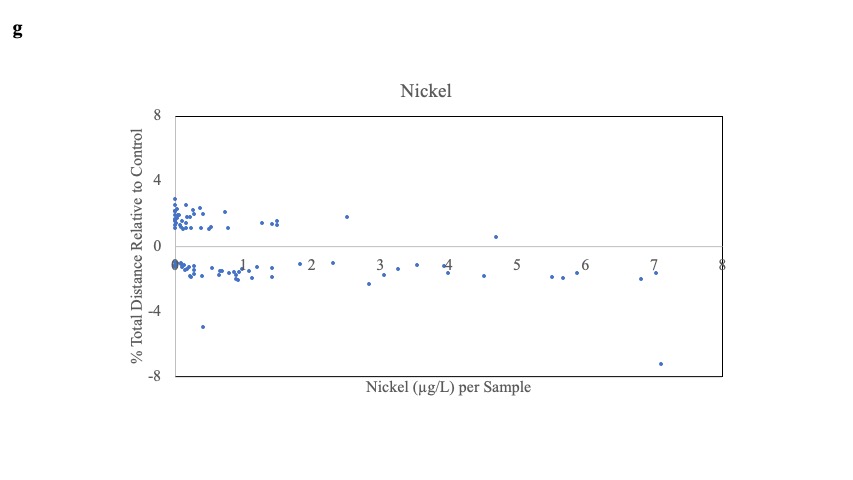

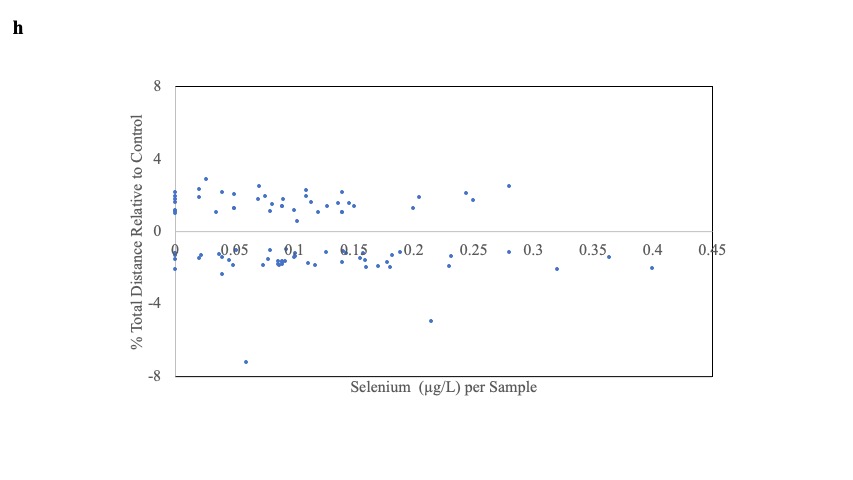
**

Figure SII: Dot plot representing total distance after a 1:1 sample / egg water exposure as a percent of 100% egg water control against the amount of A) antimony (µg/L), B) barium (µg/L), C) chromium (µg/L), D) copper (µg/L), E) iron (µg/L), F) manganese (µg/L), G) nickel (µg/L), and H) selenium (µg/L), present in a given sample. Samples that induced significant hyper or hypoactivity can be found in Table SI, p-value < 0.05, ANOVA, n=24.

**Figure SIII**

**
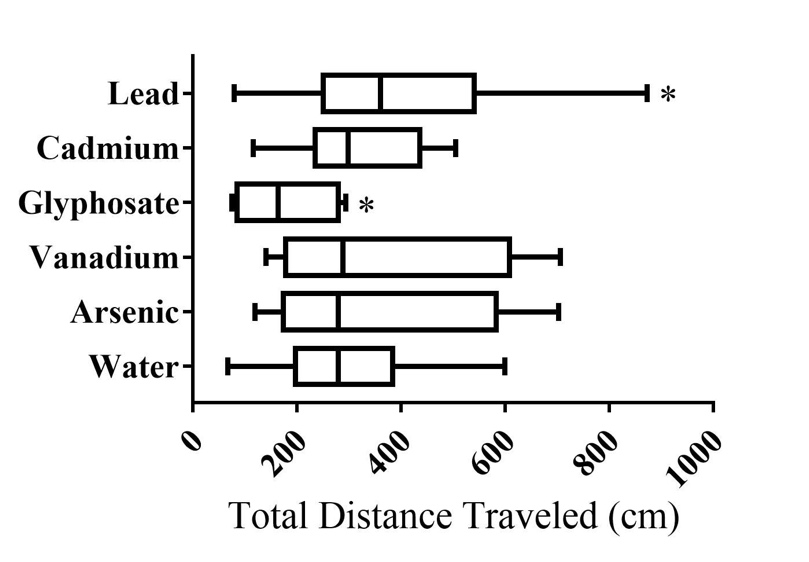
**

Figure SIII: Box and whisker plot showing distribution of TD travelled by individual zebrafish at 5 dpf after exposure to arsenic (4 μg/L), vanadium (15 μg/L), glyphosate (10 μg/L), cadmium (2 μg/L), or lead (5 μg/L) supplemented to egg water from 24 hpf to 5 dpf.  The box plot depicts the range, 1st and 4th quartile, and average. Those exposed to water represent control embryos that were reared in egg water. Asterisks indicate a significant difference in TD travelled between exposure group and control, one-way Anova LSD Fishers test p*–value < 0.05.*
