# Supplementary material for: Defining drinking water metal contaminant mixture risk by coupling zebrafish behavioral analysis with citizen science
Source: Sci Rep. 2021 Aug 27;11:17303. doi: 10.1038/s41598-021-96244-4 (PMC8397788; doi:10.1038/s41598-021-96244-4)
Supplement: Supplementary file 2 — Supplementary Table S1. [file 41598_2021_96244_MOESM2_ESM.docx]

**Defining Drinking Water Metal Contaminant Mixture Risk by Coupling Zebrafish Behavioral Analysis with Citizen Science**

*Remy Babich^1^, Emily Craig^2^, Abigail Muscat^2^, Jane Disney^3^, Anna Farrell^3^, Linda Silka^4^, Nishad Jayasundara^5^

**Affiliations:**

^1^Department of Molecular and Biomedical Sciences, University of Maine, Orono, ME

04469 US.

^2^School of Marine Sciences, University of Maine, Orono, ME 04469 US.

^3^MDI Biological Laboratory, Salisbury Cove, ME 04609 US.

^4^Senior Fellow, Senator George J. Mitchell Center for Sustainability Solutions,

University of Maine, Orono, ME 04469 US.

^5^The Nicholas School of the Environment, Duke University, Durham, NC 27708 US.

Table S1: Summary of metal analysis of a given well sample and the effect of exposure on zebrafish larval mortality, hatching inhibition rate, and total distance (TD) traveled. Fish were exposed to 50% well water sample: egg water solution from 24 hours post fertilization (hpf) to 5 days post fertilization (dpf). Three categories of data are presented depicting samples that produced no significant change (control-like), a significant decrease (hypoactive), or a significant increase (hyperactive) in TD traveled relative to 100% egg water controls (p-value < 0.05, n=24). Chemical composition of As, Cd, Pb, and U (μg/L) of a given sample are also included. These data can be visualized in Figure 1. For the 14 chemical panel of each sample, see supplemental material Table S2.

| Sample | As (μg/L) | Cd (μg/L) | | Pb (μg/L) | U (μg/L) | TD (mm) | % mortality | % chorion |  |
| --- | --- | --- | --- | --- | --- | --- | --- | --- | --- |
| Egg water control | 0.00 | | 0.00 | 0.00 | 0.00 | 7535.11 | 1.70 | 0.00 |  |
| Control-like: No significant change in TD relative to egg water control | | | | | | | | | |
| 479 | 0.10 | | 0.00 | 0.48 | 0.05 | 8855.29 | 0.02 | 0.00 |  |
| 487 | 717.91 | | 0.00 | 0.51 | 0.34 | 8930.76 | 0.07 | 0.00 |  |
| 489 | 0.12 | | 0.08 | 1.07 | 0.28 | 6223.95 | 0.08 | 0.25 |  |
| 504 | 2.44 | | 0.00 | 0.09 | 8.49 | 6054.51 | 0.00 | 0.09 |  |
| 506 | 1.57 | | 0.24 | 0.31 | 42.58 | 3864.54 | 0.91 | 0.00 |  |
| 516 | 0.74 | | 0.00 | 0.08 | 0.01 | 5424.11 | 0.07 | 0.00 |  |
| 517 | 1.27 | | 0.05 | 2.01 | 4.19 | 6053.60 | 0.53 | 0.42 |  |
| 518 | 0.96 | | 0.00 | 0.14 | 81.01 | 5451.83 | 0.02 | 0.00 |  |
| 522 | 4.95 | | 0.01 | 1.36 | 23.17 | 7042.62 | 0.02 | 0.00 |  |
| 526 | 0.26 | | 0.00 | 0.03 | 0.22 | 7685.84 | 0.00 | 0.00 |  |
| 841 | 6.23 | | 0.00 | 0.67 | 2.08 | 6555.46 | 0.00 | 0.11 |  |
| 894 | 6.73 | | 0.00 | 0.01 | 5.63 | 9515.57 | 0.03 | 0.00 |  |
| 895 | 24.11 | | 0.00 | 0.03 | 0.02 | 9461.67 | 0.00 | 0.00 |  |
| 897 | 0.03 | | 0.01 | 0.58 | 0.01 | 8108.86 | 0.36 | 0.09 |  |
| 904 | 4.31 | | 0.00 | 0.04 | 30.42 | 6360.82 | 0.04 | 0.00 |  |
| 908 | 0.52 | | 0.00 | 0.07 | 0.52 | 7008.03 | 0.00 | 0.00 |  |
| 909 | 0.40 | | 0.00 | 0.54 | 0.28 | 6953.07 | 0.04 | 0.00 |  |
| 1230 | 0.07 | | 0.01 | 0.11 | 0.96 | 8630.19 | 0.07 | 0.00 |  |
| 1237 | 0.10 | | 0.02 | 2.04 | 26.32 | 7729.85 | 0.18 | 0.00 |  |
| 1258 | 0.11 | | 0.07 | 7.14 | 0.48 | 5594.31 | 0.36 | 0.51 |  |
| 1260 | 3.64 | | 0.00 | 0.14 | 0.66 | 5618.73 | 0.02 | 0.73 |  |
| 1264 | 0.43 | | 0.02 | 0.30 | 0.25 | 8193.32 | 0.07 | 0.09 |  |
| 1270 | 0.68 | | 0.00 | 0.12 | 0.55 | 8004.28 | 0.53 | 0.31 |  |
| 1271 | 0.18 | | 0.01 | 1.25 | 69.07 | 6204.89 | 0.53 | 0.31 |  |
| 1272 | 0.40 | | 0.01 | 0.11 | 8.08 | 5627.45 | 0.00 | 0.00 |  |
| 1541 | 0.18 | | 0.01 | 0.21 | 0.05 | 8048.06 | 0.31 | 0.28 |  |
| 1544 | 0.10 | | 0.06 | 6.55 | 0.03 | 3960.31 | 0.00 | 0.02 |  |
| 1554 | 0.52 | | 0.10 | 0.31 | 0.67 | 6525.81 | 0.89 | 0.04 |  |
| 1555 | 0.08 | | 0.01 | 0.69 | 3.40 | 5858.85 | 0.04 | 0.00 |  |
| 1556 | 8.87 | | 0.01 | 0.46 | 0.14 | 8230.31 | 0.16 | 0.00 |  |
| 1561 | 1.88 | | 0.00 | 0.07 | 2.87 | 7408.15 | 0.02 | 0.00 |  |
| 1598 | 0.09 | | 0.00 | 0.20 | 2.31 | 6131.45 | 0.07 | 0.02 |  |
| Hypoactive: Significant decrease in TD relative to egg water control | | | | | | | | |  |
| 481 | 0.00 | | 0.01 | 5.57 | 0.12 | 4908.57 | 0.33 | 0.56 |  |
| 500 | 0.00 | | 0.01 | 0.78 | 5.58 | 3973.08 | 0.00 | 0.09 |  |
| 505 | 1.08 | | 0.03 | 0.76 | 210.81 | 3795.27 | 0.04 | 0.93 |  |
| 507 | 5.26 | | 0.01 | 4.07 | 629.40 | 5255.01 | 0.02 | 0.60 |  |
| 508 | 2.20 | | 0.41 | 4.74 | 15.43 | 4441.57 | 0.62 | 0.27 |  |
| 510 | 0.00 | | 0.00 | 1.89 | 0.26 | 4338.62 | 0.00 | 0.22 |  |
| 511 | 0.07 | | 0.19 | 0.38 | 0.03 | 3619.22 | 0.04 | 0.07 |  |
| 512 | 2.85 | | 0.02 | 0.71 | 4.94 | 4642.59 | 0.02 | 0.58 |  |
| 513 | 0.43 | | 0.04 | 8.16 | 14.50 | 3767.88 | 0.64 | 0.36 |  |
| 515 | 59.39 | | 0.00 | 0.17 | 2.13 | 4196.32 | 0.00 | 0.00 |  |
| 519 | 0.08 | | 0.01 | 0.38 | 2.07 | 4398.03 | 0.16 | 0.69 |  |
| 520 | 4.46 | | 0.00 | 0.27 | 388.17 | 3635.68 | 0.00 | 0.24 |  |
| 524 | 0.03 | | 0.20 | 12.16 | 0.01 | 3963.78 | 0.40 | 0.49 |  |
| 755 | 0.08 | | 0.07 | 1.12 | 0.10 | 1511.76 | 0.04 | 0.27 |  |
| 890 | 0.24 | | 0.00 | 0.49 | 0.09 | 5085.91 | 0.14 | 0.39 |  |
| 891 | 0.06 | | 0.02 | 3.77 | 0.02 | 5142.79 | 0.22 | 0.28 |  |
| 896 | 0.08 | | 0.01 | 0.59 | 0.04 | 4495.62 | 0.22 | 0.64 |  |
| 900 | 0.09 | | 0.28 | 3.04 | 0.04 | 3891.62 | 0.31 | 0.67 |  |
| 902 | 0.31 | | 0.00 | 0.03 | 5.82 | 5282.96 | 0.00 | 0.27 |  |
| 903 | 0.09 | | 0.01 | 1.38 | 0.02 | 3156.87 | 0.02 | 0.93 |  |
| 906 | 0.09 | | 0.02 | 0.90 | 0.00 | 3568.68 | 0.62 | 0.18 |  |
| 907 | 0.33 | | 0.14 | 20.09 | 0.07 | 1036.05 | 0.47 | 0.33 |  |
| 910 | 5.07 | | 0.00 | 0.45 | 30.42 | 5102.11 | 0.31 | 0.38 |  |
| 911 | 0.04 | | 0.00 | 0.82 | 0.02 | 4172.73 | 0.31 | 0.40 |  |
| 1248 | 0.74 | | 0.02 | 4.75 | 0.49 | 4139.45 | 0.58 | 0.04 |  |
| 1249 | 0.08 | | 0.01 | 11.83 | 0.02 | 4755.77 | 0.07 | 0.00 |  |
| 1250 | 7.51 | | 0.03 | 0.93 | 86.24 | 4516.01 | 0.56 | 0.42 |  |
| 1259 | 0.06 | | 0.07 | 5.73 | 0.01 | 4204.23 | 0.11 | 0.27 |  |
| 1261 | 0.18 | | 0.03 | 5.36 | 0.06 | 4007.04 | 0.00 | 0.00 |  |
| 1265 | 0.27 | | 0.01 | 0.34 | 0.03 | 4595.61 | 0.00 | 0.00 |  |
| 1267 | 0.07 | | 0.06 | 3.12 | 0.70 | 4852.35 | 0.00 | 0.36 |  |
| Hyperactive: Significant increase in TD relative to egg water control | | | | | | | | |  |
| 482 | 0.28 | | 0.00 | 0.04 | 0.40 | 13465.28 | 0.16 | 0.00 |  |
| 485 | 8.17 | | 0.00 | 0.10 | 1.35 | 14699.09 | 0.02 | 0.00 |  |
| 490 | 11.16 | | 0.00 | 0.26 | 1.04 | 16170.37 | 0.00 | 0.00 |  |
| 492 | 2.18 | | 0.00 | 0.07 | 52.49 | 11467.34 | 0.00 | 0.00 |  |
| 496 | 0.30 | | 0.02 | 0.29 | 2.42 | 14403.15 | 0.00 | 0.00 |  |
| 498 | 1.17 | | 0.00 | 0.10 | 2.91 | 16274.85 | 0.00 | 0.00 |  |
| 501 | 13.69 | | 0.00 | 0.03 | 0.94 | 13212.47 | 0.00 | 0.00 |  |
| 502 | 0.27 | | 0.01 | 0.66 | 7.76 | 10408.18 | 0.00 | 0.00 |  |
| 509 | 1.45 | | 0.03 | 9.60 | 9.91 | 10243.90 | 0.00 | 0.00 |  |
| 514 | 25.00 | | 0.00 | 0.09 | 4.40 | 10581.52 | 0.00 | 0.02 |  |
| 840 | 6.44 | | 0.00 | 0.19 | 2.15 | 14229.56 | 0.00 | 0.00 |  |
| 847 | 2.51 | | 0.00 | 0.03 | 4.69 | 9657.94 | 0.06 | 0.00 |  |
| 858 | 5.67 | | 0.00 | 0.05 | 0.62 | 11916.76 | 0.04 | 0.02 |  |
| 867 | 8.85 | | 0.00 | 0.02 | 10.68 | 11460.87 | 0.04 | 0.00 |  |
| 892 | 0.13 | | 0.00 | 0.07 | 0.26 | 16949.24 | 0.00 | 0.00 |  |
| 893 | 0.52 | | 0.10 | 1.02 | 0.17 | 13494.12 | 0.53 | 0.11 |  |
| 898 | 0.71 | | 0.00 | 0.04 | 9.88 | 17504.06 | 0.04 | 0.00 |  |
| 901 | 0.12 | | 0.00 | 0.10 | 13.00 | 12958.65 | 0.02 | 0.00 |  |
| 950 | 11.66 | | 0.00 | 0.07 | 1.31 | 10332.80 | 0.09 | 0.00 |  |
| 1226 | 0.13 | | 0.01 | 0.78 | 0.03 | 14724.24 | 0.07 | 0.00 |  |
| 1228 | 6.70 | | 0.00 | 0.15 | 3274.37 | 18890.70 | 0.02 | 0.00 |  |
| 1229 | 0.05 | | 0.02 | 0.31 | 0.23 | 16348.05 | 0.04 | 0.00 |  |
| 1238 | 1.61 | | 0.02 | 0.10 | 13.64 | 12274.28 | 0.07 | 0.00 |  |
| 1245 | 14.91 | | 0.00 | 1.84 | 0.28 | 15390.33 | 0.11 | 0.00 |  |
| 1247 | 1.31 | | 0.00 | 0.20 | 5.54 | 15938.81 | 0.00 | 0.00 |  |
| 1252 | 0.56 | | 0.00 | 0.03 | 41.45 | 14170.24 | 0.02 | 0.00 |  |
| 1523 | 1.16 | | 0.01 | 0.18 | 31.11 | 18955.95 | 0.00 | 0.19 |  |
| 1548 | 8.60 | | 0.00 | 0.08 | 1.48 | 11334.50 | 0.00 | 0.00 |  |
| 1589 | 2.70 | | 0.00 | 0.10 | 0.05 | 21595.08 | 0.00 | 0.00 |  |
