# Supplementary material for: Defining drinking water metal contaminant mixture risk by coupling zebrafish behavioral analysis with citizen science
Source: Sci Rep. 2021 Aug 27;11:17303. doi: 10.1038/s41598-021-96244-4 (PMC8397788; doi:10.1038/s41598-021-96244-4)
Supplement: Supplementary file 4 — Supplementary Information 1. [file 41598_2021_96244_MOESM4_ESM.docx]

**Defining Drinking Water Metal Contaminant Mixture Risk by Coupling Zebrafish Behavioral Analysis with Citizen Science**

*Remy Babich^1^, Emily Craig^2^, Abigail Muscat^2^, Jane Disney^3^, Anna Farrell^3^, Linda Silka^4^, Nishad Jayasundara^5^

**Affiliations:**

^1^Department of Molecular and Biomedical Sciences, University of Maine, Orono, ME

04469 US.

^2^School of Marine Sciences, University of Maine, Orono, ME 04469 US.

^3^MDI Biological Laboratory, Salisbury Cove, ME 04609 US.

^4^Senior Fellow, Senator George J. Mitchell Center for Sustainability Solutions,

University of Maine, Orono, ME 04469 US.

^5^The Nicholas School of the Environment, Duke University, Durham, NC 27708 US.

SUPPLEMENTAL METHODS: SPSS PCA SYNTAX

FACTOR

/VARIABLES Be Cr Mn Fe Ni Cu As Se Cd Sb Ba Tl Pb U (OR ANY COMBINATION OF METALS)

/MISSING LISTWISE

/ANALYSIS Be Cr Mn Fe Ni Cu As Se Cd Sb Ba Tl Pb U (OR ANY COMBINATION OF METALS)

/MISSING LISTWISE

/PRINT INITIAL CORRELATION KMO EXTRACTION ROTATION FSCORE

/PLOT ROTATION

/CRITERIA FACTORS(2) ITERATE(25)

/EXTRACTION PC

/CRITERIA ITERATE(25) DELTA(0)

/ROTATION OBLIMIN

/SAVE REG(ALL)

/METHOD=CORRELATION.
